# Qualitative Research With HIV+ Older Adults Living in Rural Areas: Methodological Considerations During COVID-19

**DOI:** 10.1093/geroni/igab046.2241

**Published:** 2021-12-17

**Authors:** Erin Robinson

**Affiliations:** University of Missouri, Columbia, Missouri, United States

## Abstract

Older adults living with HIV/AIDS (OALWHA) in rural areas of the U.S. are a highly marginalized community. Intersectional stigma related to age, HIV status, geography, sexual orientation, gender identity, and race oftentimes create a complex lived experience for this population group. While there is a significant need for qualitative research that highlights the intersecting stigmas experienced by OALWHA in rural areas, recruitment challenges exist. Fear of being outed in their rural communities, due to their HIV status and LGBTQ+ identities, makes many OALWHA reluctant to participate in research. However, there is much resiliency in the population as well, especially during the COVID-19 pandemic. In fact, as research approaches have pivoted to phone/virtual data collection during the pandemic, this can help promote anonymity among this population group. This presentation will detail methodological considerations for recruitment, data collection, and analysis for qualitative research with OALWHA in rural areas of the U.S.

